# A privacy-preserving HLA imputation method with homomorphic encryption

**DOI:** 10.1016/j.isci.2025.113442

**Published:** 2025-08-26

**Authors:** Hakin Kim, Intak Hwang, Yongsoo Song, Buhm Han

**Affiliations:** 1 Interdisciplinary Program in Bioengineering, Seoul National University, Seoul, Republic of Korea; 2 Department of Computer Science and Engineering, Seoul National University, Seoul, Republic of Korea; 3 Convergence Dementia Research Center, Seoul National University Medical Research Center, Seoul, Republic of Korea; 4 Department of Biomedical Sciences, BK21 Plus Biomedical Science Project, Seoul National University College of Medicine, Seoul, Republic of Korea

**Keywords:** Genetics, Immunology, Data encryption

## Abstract

In recent years, several HLA imputation methods on local servers have been developed, using single nucleotide polymorphisms (SNPs) as inputs to predict Human Leukocyte Antigen (HLA) genotypes. However, these methods require HLA reference panels, which are often unavailable and memory-intensive. Cloud-based outsourced HLA imputation overcomes these limitations by utilizing built-in reference panels on the cloud server, removing local storage needs. However, uploading genotype data online raises privacy concerns. Although secure from third parties, the uploaded data can be misused by cloud server administrators. Additionally, reference panels and their HLA imputation model on the cloud servers must be safeguarded against malicious clients. To address these privacy issues, we developed privateHLA, the first secure HLA imputation method using homomorphic encryption. privateHLA securely performs HLA imputation on an outsourced server, protecting both the client’s data and enhancing model privacy. privateHLA outperformed SNP2HLA but had slightly lower accuracy than CookHLA, both of which are plaintext-based methods.

## Highlights

• Cloud-based HLA imputation offers convenience but raises privacy concerns
• PrivateHLA is the first secure HLA imputation method using homomorphic encryption
• PrivateHLA protects client data and model privacy on outsourced servers
• PrivateHLA is comparable to existing plaintext methods

## Graphical abstract

**Figure:**
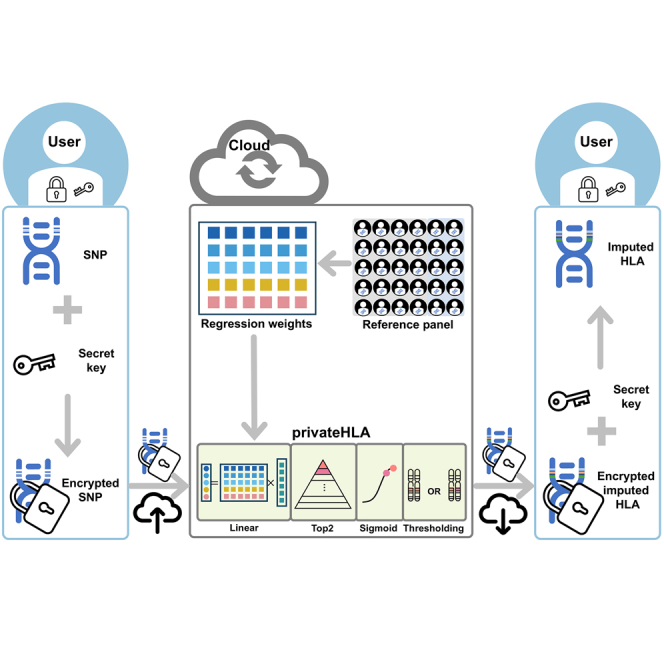

## Introduction

Human Leukocyte Antigen (HLA) molecules are produced by genes at 6p21.3 with spanning approximately 5 Mb in length and regulate the immune system.^1^ The structure of HLA is more complex than the other regions due to the high level of polymorphism and extensive linkage disequilibrium. HLA has a significant impact on drug reactions and organ transplantation, particularly hematopoietic stem cell transplantation. For patients, identifying an individual’s HLA genotypes is crucial to achieving precision medicine. For researchers, identifying samples’ HLA genotypes is crucial for fine-mapping analysis to comprehend the immune-related mechanism. However, classical HLA typing is expensive, making it challenging to obtain every HLA genotype within a cost limit. To overcome this cost limitation, several HLA imputation methods have been developed.^2^ HLA imputation utilizes a reference panel composed of SNPs and HLA genotypes.

There are two ways to perform HLA imputation: locally on a user’s computer and on an outsourced server (cloud server).^3^ Local imputation has the advantage that the genotype data is securely protected. However, local imputation requires lots of laborious efforts including reference panel preparation. Local imputation normally requires the user to request access to the HLA reference panel, which is necessary for HLA imputation. However, most HLA reference panels are restricted to research use only, not for recreational or commercial purposes. Moreover, the user must phase the panel if it is not already phased. Here, phasing is a procedure that organizes genotype data into continuous haplotype sequences; it is time-consuming but essential for accurate imputation. Widely used local imputation methods are SNP2HLA^4^ and CookHLA.^5^ On the other hand, outsourcing HLA imputation requires the user to upload SNP genotype data to a server. This approach is convenient in that it does not require an HLA reference panel and memory space for users. However, outsourcing HLA imputation poses privacy risks, as outsourcing potentially exposes the data to the server administrator, even though the data remains concealed from third parties.

To build a safe and secure platform for the HLA imputation on an outsourced server, two major privacy tasks must be addressed. First, the user’s SNP data and imputed HLA data must be protected from the server’s administrators. Below are the reasons why they must be protected. While SNP data is already handled cautiously due to its ability to reveal individual identity and familial relationships using only a small number of loci,^6^ HLA genotypes raise serious concerns, too. Specifically, European G29 defines sensitive data based on singularity, correlation, and inference. HLA data fulfills all three criteria and is therefore classified as identifiable personal health information (PHI).^7^^,^^8^^,^^9^ Its high polymorphism enables individual-level identification (singularity); it is strongly associated with clinical conditions such as autoimmune diseases, drug reactions, and transplant compatibility (correlation)^9^; and certain HLA alleles enable the inference of disease susceptibility, genetic ancestry, and familial relationships (inference).^10^ These characteristics justify the classification of HLA genotypes as highly sensitive personal information requiring robust privacy protection. The need to protect the sensitive genetic data from administrators has been further underscored by real-world incidents—for example, a direct-to-consumer genetic testing company’s sharing of customer data with a pharmaceutical firm for research purposes, which raised significant concerns over transparency, consent, and the commercial use of sensitive genomic data.^11^

Second, the imputation reference panels and models themselves must be protected against misuse. Below are the reasons why they must be protected. Most HLA reference panels are proprietary and not publicly available, and the models built upon them often encode population-specific genomic features. Recent studies have demonstrated that such models are vulnerable to reconstruction attacks, which can enable adversaries to infer private genomic content from imputation outputs.^12^ These findings underscore the need for comprehensive privacy-preserving strategies that not only secure user data but also safeguard the integrity of the imputation pipeline itself. Homomorphic encryption provides a promising solution by allowing secure computation directly on encrypted inputs, protecting both the data and the models throughout the entire imputation process.

To address these privacy concerns, we developed privateHLA, the first outsourced HLA imputation model with homomorphic encryption. A notable feature of our method is that we employ a nonlinear function within homomorphic encryption, specifically logistic regression, to securely perform HLA imputation. As a result, privateHLA offers two major advantages. First, it protects both inputs (SNP genotypes) and outputs (predicted HLA genotypes) from the server personnel. Second, privateHLA enhances model privacy against malicious server users by employing nonlinear functions in homomorphic encryption.

Unlike existing SNP imputation models^13^^,^^14^^,^^15^^,^^16^ using homomorphic encryption—which typically adopt simplified mathematical formulations due to the high computational cost of homomorphic encryption—privateHLA is designed to homomorphically evaluate both linear and nonlinear functions to enhance model privacy. This allows privateHLA to return only the indices of the imputed allele pair for each gene, rather than all probability values for possible alleles. This approach makes it more difficult for malicious users to attack the model. By contrast, existing homomorphic encryption-based SNP imputation methods^15^^,^^16^ only homomorphically evaluate the linear function and return probabilities for all alleles. This approach makes these models vulnerable to reconstruction attacks.^12^^,^^17^^,^^18^ Additionally, another homomorphic encryption-based SNP imputation method utilized a look-up table that includes combinations of tag SNPs and outputs probabilities.^19^ However, this method is vulnerable to membership inference attacks. Consequently, these homomorphic encryption-based imputation methods^15^^,^^16^^,^^19^ must rely on publicly available reference panels due to their vulnerable model privacy.

Homomorphic encryption encompasses several schemes, notably BFV (Brakerski/Fan-Vercauteren),^20^ CKKS (Cheon-Kim-Kim-Song),^21^ and TFHE (Torus Fully Homomorphic Encryption),^22^ each optimized for different encrypted computations. Leveled schemes, such as BFV and CKKS, are designed for batched evaluations of encrypted data, making them suitable for applications that require high throughput. However, they can usually only evaluate arithmetic operations such as additions and multiplications, so non-arithmetic operations such as comparison should be approximated, which results in huge performance overhead. Meanwhile, TFHE operates on individual bits of data, allowing basic logical operations such as AND, OR, and NOT to be performed directly on encrypted bits without decrypting them. TFHE provides high precision and minimal latency (delay). TFHE excels at processing small data and can compute arbitrary functions efficiently using a look-up table, which is a precomputed table of input and output values of a given function. We chose TFHE due to its effectiveness for evaluating nonlinear functions. privateHLA showed sufficiently low error rates even with low-precision computation.

We separately imputed HLA genotypes for 88 Europeans^23^ and 413 Koreans,^24^ comparing the results of privateHLA with classical methods in plaintext (CookHLA and SNP2HLA). We used 5,225 European reference samples^25^ for the European dataset and 9,773 Chinese reference samples^26^ for the Korean dataset. Despite performing calculations in an encrypted state that allows error for security purposes, privateHLA demonstrated accuracy comparable to SNP2HLA and CookHLA. In the European dataset, privateHLA achieved an average accuracy of 94.79% for all HLA genes, while CookHLA achieved 95.36% and SNP2HLA achieved 93.09%. Similarly, in the Korean dataset, privateHLA achieved an average accuracy of 93.66%, while CookHLA achieved 95.38% and SNP2HLA achieved 92.11%. Overall, the accuracy of privateHLA was higher than SNP2HLA but lower than CookHLA.

## Results

### Practicality of privateHLA workflow

The motivations behind privateHLA involve the interaction between a user and an outsourced cloud server, which serves as the HLA imputation service provider. The user, who may be a researcher, clinician, or hematopoietic stem cell donation candidate, has SNP genotype data but lacks an HLA reference panel. The user aims to impute HLA genotypes without exposing their SNP genotypes or the predicted HLA genotypes to the cloud server. Meanwhile, the cloud server, having trained an HLA imputation model from a non-public HLA reference panel, seeks to secure the model. To address these concerns, privateHLA safeguards both SNP genotype inputs and predicted HLA genotype outputs, while enhancing model privacy.

**Figure 1:**
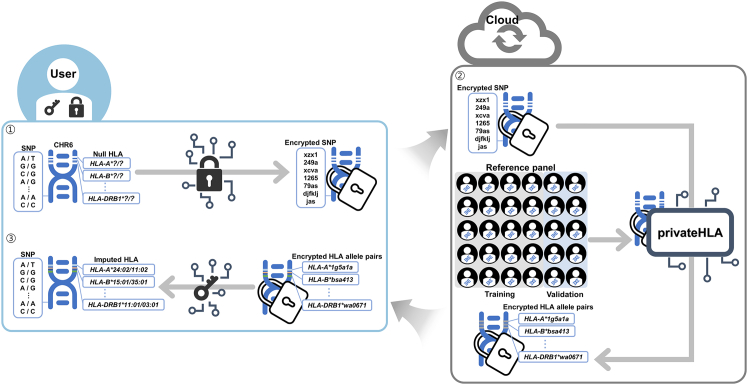
Workflow of how privateHLA works in practice The user generates a pair of keys: a secret key and a public key. The secret key is used for encryption and decryption, while the public key enables encrypted computation on the server. After encrypting the SNP genotype data, the user uploads it to the outsourced cloud server. The server uses the encrypted SNP data to predict the most probable HLA allele pairs, which are then returned to the user in encrypted form. Finally, the user decrypts the allele pairs.

privateHLA employs a homomorphic encryption scheme to protect user information (Figure 1). First, the user generates a pair of keys: a secret key and a public key. Using the secret key, the user encrypts SNP genotype data and uploads it to the cloud server along with the public key. The server, equipped with a trained HLA imputation model, uses the public key to predict HLA genotypes from the encrypted SNP data. It then returns the encrypted indices of the most probable allele pairs, leveraging nonlinear functions inherent in homomorphic encryption. Upon receiving these encrypted indices, the user decrypts them with the secret key to obtain the imputed HLA alleles.

In more detail, the secret key is crucial for both encrypting SNP data and decrypting HLA data from the imputation. And the public key facilitates the computation on the encrypted data and serves as the bootstrapping key in the TFHE scheme. The user is responsible for generating and managing both keys, keeping the secret key strictly private. The public key can be shared with the cloud server for computations without compromising privacy. This key management ensures that only the user can decrypt and access sensitive genotype data, maintaining control over data usage even when processed by an outsourced cloud server. Detailed information regarding the keys is provided in the STAR Methods.

privateHLA implements end-to-end homomorphic encryption to securely utilize HLA reference panels on an outsourced server, and differs from previous approaches. Previous homomorphic encryption-based SNP imputation models have limitations^16^; they are convex and only encrypt the linear part of the model, returning probabilities for all alleles, which poses privacy risks. Convex models are vulnerable to reconstruction attacks, especially when all probabilities are disclosed.^27^^,^^28^ This vulnerability arises from the exposure of these probabilities, as they can inadvertently reveal model weights and risk exposing the training data. By contrast, privateHLA comprehensively encrypts both the linear and nonlinear parts. This allows the outsourced server to return only two indices of the most probable allele pair, withholding probabilities for all alleles. For example, if *HLA-B* has 99 possible alleles, privateHLA returns only the indices of the imputed allele pair (such as *HLA-B∗15:01* and *HLA-B∗51:01*) for a sample, rather than 99 probabilities. Furthermore, privateHLA includes a threshold parameter for allele determination, which increases model complexity (details in the STAR Methods). These approaches enhance model privacy, particularly for reference panels that are usually unavailable to the public.

In summary, the motivation behind privateHLA (the problem privateHLA addresses) and its proposed solution are as follows.

(Motivation 1) The user wants to impute HLA genotypes on an outsourced server without any privacy concerns, even from the server’s administrator.
(Solution 1) privateHLA protects user’s SNPs and imputed HLA genotypes by using homomorphic encryption.
(Motivation 2) The outsourced server houses a valuable HLA reference panel and imputation model that needs to be kept secure.
(Solution 2) privateHLA applies not only linear functions but also nonlinear functions in homomorphic encryption, returning only the indices of the imputed pair.

The following example demonstrates the application of privateHLA in clinical research. HLA typing is crucial for identifying candidates for HLA-restricted immunotherapies and patient stratification.^29^ However, its high cost often limits its large-scale application. Clancy et al.^30^ showed that HLA genotypes can be effectively imputed from SNP data to identify HLA homozygous individuals, facilitating their use in cell therapy and immunogenetic research. Outsourced imputation services, such as the Michigan Imputation Server and the SHLARC platform, provide convenient solutions for HLA imputation.^31^^,^^32^ Nonetheless, privacy regulations frequently restrict the uploading of raw genomic data to external servers. privateHLA addresses this challenge by enabling the local encryption of SNP data using a secret key, securely stored within the hospital. A corresponding public key, also known as the bootstrapping key in the TFHE scheme, is uploaded to the cloud along with the encrypted SNP data. This setup allows cloud servers to perform computations directly on the encrypted genotypes without accessing plaintext data. Only the hospital possessing the secret key can decrypt the results, ensuring complete data privacy. This configuration enables clinician-scientists to incorporate imputed HLA types into biomarker discovery, trial design, or patient stratification workflows while adhering to regulatory compliance.

### Accuracy comparison across genes

We compared the accuracy of privateHLA with two representative HLA imputation methods in plaintext, SNP2HLA^4^ and CookHLA.^5^ We imputed HapMap European (*N* = 88)^23^ and Korean (*N* = 413)^24^ target panels using T1DGC (*N* = 5,225)^25^ and HAN Chinese (*N* = 9,773)^26^ reference panels, respectively. We considered 8 HLA genes (*HLA-A*, *-B*, *-C*, *-DPA1*, *-DPB1*, *-DQA1*, *-DQB1* and *-DRB1*), and only those genes present in both the reference panel and the target data were imputed in the analysis.

**Figure 2:**
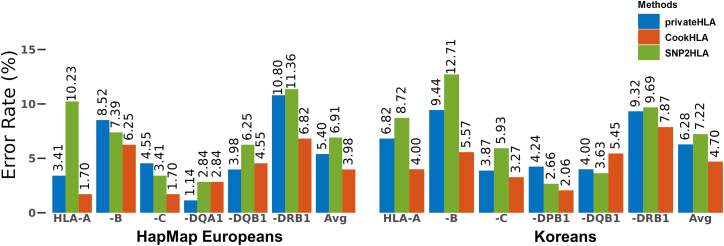
Error rates of privateHLA, CookHLA and SNP2HLA We compared the error rates of privateHLA with those of CookHLA and SNP2HLA, two representative HLA imputation methods, in plaintext for the HapMap European and Korean target panels. privateHLA exhibited comparable error rates to the plaintext methods, with accuracy higher than SNP2HLA and lower than CookHLA.

privateHLA’s error rates were comparable to those of existing methods in plaintext (Figure 2). privateHLA achieved higher accuracy than SNP2HLA but lower accuracy than CookHLA. For the HapMap European target panel, privateHLA had an average error rate of 5.40%, outperforming SNP2HLA’s 6.91% but falling short of CookHLA’s 3.98%. In terms of specific HLA genes, privateHLA performed better than SNP2HLA for *HLA-A, -DQA1, -DQB1,* and -DRB1, while privateHLA performed more poorly than SNP2HLA for *HLA-B* and *-C*. For instance, for *HLA-A*, privateHLA’s error rate was 3.41%, while the error rates were 1.70% for CookHLA and 10.23% for SNP2HLA. For *HLA-B*, privateHLA’s error rate was 8.52%, slightly worse than both 6.25% for CookHLA and 7.39% for SNP2HLA. Notably, privateHLA excelled in imputing *HLA-DQA1*, having an error rate of 1.14%, while both CookHLA and SNP2HLA had 2.84%. For the Korean target panel, privateHLA had an average error rate of 6.28%, outperforming SNP2HLA’s 7.22% but falling short of CookHLA’s 4.70%. Overall, privateHLA maintained lower error rates than SNP2HLA, except for *HLA-DPB1*.

Additionally, we compared the prediction accuracies of privateHLA, which performs logistic regression on encrypted data, and plain privateHLA, a non-encrypted version that uses the same model and parameters. Both models achieved nearly identical accuracies, which means that the accuracy loss due to the encryption is negligible (Figure S1).

### Accuracy comparison across allele frequencies

**Figure 3:**
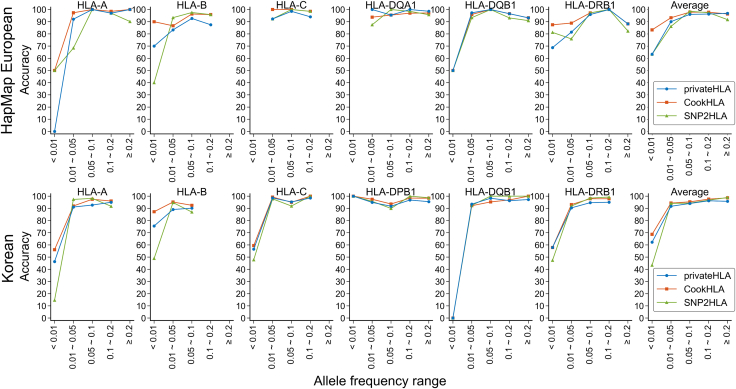
Comparison of imputation accuracies across the minor allele frequency bins We calculated HLA minor allele frequency in each reference panel. With this frequency, we grouped the alleles into 5 bins. In each bin, we calculated the imputation accuracy. HLA allele pairs, which are then returned to the user in encrypted form. Finally, the user decrypts the allele pairs.

We calculated the imputation accuracies of privateHLA, SNP2HLA, and CookHLA across various minor allele frequency (MAF) bins (Figure 3). To start, HLA alleles were grouped into five categories based on their frequencies in each reference panel: (1)MAF<0.01,(2)0.01≤MAF<0.05,(3)0.05≤MAF<0.1,(4)0.1≤MAF<0.2and(5)0.2≤MAF. Accuracy was then calculated as the proportion of correctly imputed alleles in each bin.

In most of the MAF bins, privateHLA exhibited better accuracy than SNP2HLA but had lower accuracy than CookHLA. First, we imputed the HapMap European panel. For rare alleles with MAF<0.01, privateHLA and SNP2HLA both achieved an average accuracy of 63.3%, while CookHLA performed better with 83.3%. In contrast, for common alleles with MAF≥0.2, privateHLA outperformed both CookHLA and SNP2HLA with an average accuracy of 96.89%, slightly higher compared to 96.37% and 91.70% respectively.

Second, we imputed the Korean panel. For rare alleles with MAF<0.01, privateHLA achieved an average accuracy of 62.26%, surpassing SNP2HLA’s 43.39% but falling short of CookHLA’s 68.67%. privateHLA, despite being based on a relatively straightforward logistic regression model, outperformed the more complex SNP2HLA based on a Hidden Markov Model. This superior performance is evident even in rare alleles with MAF<0.01 when inputting the Korean panel. We observed notable differences in accuracy between methods, particularly in *HLA-A* and *-B*.

When imputing HapMap Europeans' *HLA-A* with MAF<0.01 (Figure 3), privateHLA showed 0% accuracy. This poor performance is due to its failure to predict the alleles *HLA-A∗02:04* and *HLA-A∗02:06*, both of which were observed only once in the target data. Despite a total of two occurrences in the dataset, not a single one was correctly predicted by privateHLA. In contrast, SNP2HLA and CookHLA correctly identified *HLA-A∗02:06* in one sample, achieving 50% accuracy.

### Limited availability of human leukocyte antigen panels

Table 1 summarizes currently available HLA panels from diverse studies, including their public accessibility. Some HLA panels are publicly accessible, whereas many others require formal applications for access. Regardless of public accessibility, their non-research uses are generally restricted—most such uses require additional permissions or are entirely prohibited. For example, the 1 KG,^44^ HapMap European,^23^^,^^36^ HAN Chinese,^26^ Korean,^24^ and PanAsian^40^ panels are publicly accessible, whereas the T1DGC,^25^ Estonian,^34^ Japanese,^37^^,^^38^ Jackson Heart Study,^41^ COPDGene,^42^ and Multi-ethnic Study of Atherosclerosis^43^ panels are accessible only through application.

**Table 1:** List of currently available HLA panels

Panel	Ancestry	Sample Size	Accessibility	Data Access URL
Type 1 Diabetes Genetics Consortium^25^	European	5,225	Accessible through application	https://repository.niddk.nih.gov/studies/t1dgc-special/
HapMap European^33^	European	124	Publicly accessible	https://software.broadinstitute.org/mpg/snp2hla/
Estonian^34^	European	2,244	Accessible through application	https://www.ncbi.nlm.nih.gov/pmc/articles/PMC8959399/#SD1
Finnish^35^	European	1,150	Only through HIBAG model	https://github.com/FRCBS/HLA-imputation
European^36^	European	401	Only through HIBAG model	https://github.com/zhengxwen/HIBAG/
HAN Chinese^26^	East Asian	9,773	Publicly accessible	http://gigadb.org/dataset/100156
Korean^24^	East Asian	413	Publicly accessible	https://journals.plos.org/plosone/article?id=10.1371/journal.pone.0112546
Japanese^37^^,^^38^	East Asian	1,120	Accessible through application	https://humandbs.dbcls.jp/en/hum0114-v3
Taiwanese^39^	East Asian	1,012	Only through HIBAG model & Accessible through application	https://github.com/zhengxwen/HIBAG/blob/master/vignettes/HIBAG.Rmd
PanAsian^40^	East and South Asian	530	Publicly accessible	https://software.broadinstitute.org/mpg/snp2hla/
Jackson Heart Study^41^	African American	5,306	Accessible through application	https://redcap.umc.edu/surveys/?s=R48NR37HA8
COPDGene^42^	Non-Hispanic white and African American	10,623	Accessible through application	.
Multi-Ethnic Study of Atherosclerosis^43^	Multiple Ancestries	4,620	Accessible through application	https://www.mesa-nhlbi.org/
1000 Genomes Project^44^	Multiple Ancestries	2,380	Publicly accessible	https://www.internationalgenome.org/data/

We summarized currently available HLA panels with their features including publicly accessibility. Only 1 KG, HapMap European, HAN Chinese, Korean and PanAsian panels are publicly accessible except for the HIBAG of which reference panel’s format is different from SNP2HLA and CookHLA. HIBAG’s files comprise solely compressed model parameters, excluding the raw panel data.

In addition to the panels that are publicly accessible and the panels that require an application, the third category of panels consists of panels that are exclusively provided in a specific pre-built model. HIBAG^45^ model retains parameters trained from raw reference panel data rather than providing the raw data itself. For example, the Finnish,^35^ European^36^ and Taiwanese^39^ reference panels are exclusively accessible through HIBAG models. The lack of raw HLA panel data further limits the availability of these HLA panels.

These limitations in accessibility, usage, and format inconsistencies highlight the need to safeguard the privacy of HLA reference panels on an outsourced server. To address this issue, privateHLA offers a solution to secure HLA reference panels on the server.

### Time and memory consumption

We present time and memory consumption benchmark results for privateHLA. We measured the results on a server machine equipped with an Intel Xeon Platinum 8268 CPU at 2.9 GHz. Our implementation is based on the TFHE-go library, which offers an optimized implementation of the TFHE scheme with AVX2 acceleration.^46^

Table 2 shows the time and memory consumption for the imputation of one sample in each target panel. The comparison part (Top2 function; more details in the STAR Methods) constitutes the largest portion (98.69%) of the total processing time. As expected, the Top2 function’s processing time increases with the number of HLA alleles; *HLA-B*, which has the most alleles, takes the longest. Specifically, when imputing the Korean panel, *HLA-B* and *HLA-DPA1* have 99 and 5 alleles, respectively. The Top2 function takes 20.81 s for *HLA-B*, which is 33 times longer than the 0.617 s for *HLA-DPA1*. Interestingly, the number of SNPs in the reference panel (5,754 in the European and 21,820 in the Korean panels) has a limited impact on the processing time, primarily affecting only the linear part. Sigmoid and Threshold functions take approximately 106-107 ms across genes.

**Table 2:** Time and memory consumption table of privateHLA

Gene	HapMap European						
	Function	Time		Memory		Number of Alleles	Number of SNPs
HLA-A	Linear	1.91 ms	10.60s	4.01Mi	5.06Mi	49	5,754
	Top2	10.48s		1013Ki			
	Sigmoid &Threshold	106.8 ms		72Ki			
HLA-B	Linear	3.80 ms	21.02s	7.85Mi	9.76Mi	69	
	Top2	20.88s		1.83Mi			
	Sigmoid &Threshold	106.9 ms		72Ki			
HLA-C	Linear	1.30 ms	7.13s	2.72Mi	3.50Mi	33	
	Top2	7.04s		721Ki			
	Sigmoid &Threshold	106.9 ms		72Ki			
HLA-DQA1	Linear	344.7μs	1.34s	739Ki	1.05Mi	8	
	Top2	1.26s		270Ki			
	Sigmoid &Threshold	80.17 ms		72Ki			
HLA-DQB1	Linear	731.5μs	3.91s	1.52Mi	2.03Mi	18	
	Top2	3.81s		451Ki			
	Sigmoid &Threshold	106.3 ms		72Ki			
HLA-DRB1	Linear	2.00 ms	11.05s	4.17Mi	5.26Mi	51	
	Top2	10.91s		1.02Mi			
	Sigmoid &Threshold	107.2 ms		72Ki			

Gene	Korean						
	Function	Time		Memory		Number of Alleles	Number of SNPs
HLA-A	Linear	2.52 ms	10.80s	4.09Mi	5.16Mi	51	21,820
	Top2	10.68s		1.00Mi			
	Sigmoid &Threshold	106.9 ms		72Ki			
HLA-B	Linear	5.17 ms	20.99s	7.85Mi	9.76Mi	99	
	Top2	20.81s		1.83Mi			
	Sigmoid &Threshold	106.5 ms		72Ki			
HLA-C	Linear	2.14 ms	9.07s	3.44Mi	4.38Mi	44	
	Top2	8.98s		883Ki			
	Sigmoid &Threshold	106.6 ms		72Ki			
HLA-DPA1	Linear	241.8μs	695.7 ms	410Ki	681Ki	5	
	Top2	617.3 ms		198Ki			
	Sigmoid &Threshold	79.68 ms		72Ki			
HLA-DPB1	Linear	1.13 ms	4.79s	1.84Mi	2.42Mi	23	
	Top2	4.68s		523Ki			
	Sigmoid &Threshold	106.4 ms		72Ki			
HLA-DQA1	Linear	829.9μs	3.48s	1.36Mi	1.83Mi	17	
	Top2	3.38s		414Ki			
	Sigmoid &Threshold	106.4 ms		72Ki			
HLA-DQB1	Linear	930.9μs	3.93s	1.52Mi	2.03Mi	19	
	Top2	3.81s		451Ki			
	Sigmoid &Threshold	106.1 ms		72Ki			
HLA-DRB1	Linear	2.30 ms	9.72s	3.68Mi	4.67Mi	47	
	Top2	9.64s		938Ki			
	Sigmoid &Threshold	106.7 ms		72Ki			

We calculated the time and memory for major functions of privateHLA per one sample for each group. Abbreviations: s, second; ms, millisecond; Mi, Mebibyte; Ki, Kibibyte.

privateHLA is also efficient in terms of memory consumption. In particular, it uses at most 9.76MiB for *HLA-B*, which can be considered negligible in modern computers. Note that the linear function is the most memory-intensive component, as it requires storing the encrypted probabilities for all HLA alleles after the linear operation. Not shown in Table 2, encrypted SNP ciphertexts are also considerably small, with the HapMap European being 96.05 KiB and the Korean being 352.2 KiB per SNP. The minimum system requirements to run the privateHLA model are around 150–200 MB of memory. This amount of memory is sufficient for most standard computers, so it should run smoothly on most machines without requiring high-end hardware.

## Discussion

Our novel outsourced privacy-preserving HLA imputation method, privateHLA, represents an advancement in secure genomic analysis. privateHLA protects users' sensitive genetic information (SNP genotype and predicted HLA genotype) by utilizing homomorphic encryption. privateHLA enhances model privacy by implementing nonlinear functions within homomorphic encryption. In our benchmarking analysis, privateHLA achieved accuracy comparable to plaintext HLA imputation methods even for rare HLA alleles with MAF<0.01.

The privateHLA model, based on logistic regression, has shown superior imputation accuracy compared to SNP2HLA, which uses a Hidden Markov Model. This challenges the assumption that more complex models normally perform better. For instance, Deep-HLA,^47^ built on deep learning, can be viewed as an extension of logistic regression and also substantially outperformed SNP2HLA. This suggests that simpler architectures, like privateHLA, can achieve competitive performance. Despite its lower accuracy, SNP2HLA provides additional functionalities, including dosage information and imputation quality metrics, which are valuable for downstream genomic analyses.

privateHLA returns only the indices of the most probable HLA alleles rather than their associated probabilities, mainly due to privacy considerations of the reference panel. However, in cases where the user is not considered malicious and downstream analyses require probability values, it is possible to optionally retrieve the probabilities of the most likely alleles. This allows privateHLA to support additional analytical needs, such as calculating confidence scores using the sample size of the reference panel.

Homomorphic encryption has potential to facilitate compliance with legal and ethical regulations for genetic data sharing. Regulations such as GDPR (General Data Protection Regulation) and CCPA (California Consumer Privacy Act) impose data protection requirements for genetic data sharing, often encouraging encryption.^19^^,^^48^^,^^49^^,^^50^ By enabling computations on the encrypted data, homomorphic encryption can ensure secure genetic data sharing. This, in turn, can support the construction of larger reference panels, improving imputation accuracy across methods.^51^^,^^52^ As a result, this approach could enhance the accuracy of privateHLA, potentially surpassing methods like CookHLA that rely on smaller sample sizes. This presents a promising future direction for privateHLA.

privateHLA’s Linear Function processes tens of thousands of SNPs at a rapid computational speed. Compared to existing homomorphic-encryption-based SNP imputation methods,^16^ privateHLA requires significantly more SNPs as input due to the high polymorphism and long-range linkage disequilibrium of HLA genes. When imputing *HLA-A*, for example, privateHLA processed one European sample (49 HLA alleles, 5,754 SNPs) in 1.91 ms and one Korean sample (51 HLA alleles, 21,820 SNPs) in 2.52 ms. This corresponds to 38.97 μs and 49.41 μs per allele per individual for the European and Korean panels, respectively (Table 2). The longer per-allele runtime in the Korean panel is due to the larger number of SNPs. In contrast, Kim et al.^16^ (SNU-CKKS team) evaluated only the linear function within homomorphic encryption, reporting a shorter runtime of 0.312 μs per target SNP per individual using just 30–40 SNPs. Despite processing far more SNPs than the 30–40, privateHLA’s Linear Function remains computationally efficient with microsecond-level per-allele runtime.

privateHLA’s structure can be extended to various complex imputation tasks beyond HLA. This structure can help impute complex genomic regions where traditional SNP imputation struggles due to the high number of alleles. For example, the structure can be applied to impute the KIR (Killer-cell Immunoglobulin-like Receptor) genotypes, which are vital for immune function and display extensive genetic diversity.^53^ Additionally, it can be applied to impute ABO blood types and CYP (Cytochrome P450) genotypes, the latter being important for drug metabolism.^54^ By these possible extensions, we expect that privateHLA will demonstrate its broad utility for secure outsourced genomic services based on SNP data.

Homomorphic encryption can be effectively applied not only to privateHLA, which is based on logistic regression model, but also to tools like HIBAG.^55^ Specifically, in HIBAG, encrypting the sensitive test set while keeping the reference haplotype frequencies in plaintext allows the imputation process to be performed securely. HIBAG computes posterior probabilities by summing the products of these frequencies over candidate haplotype pairs. Since the algorithm relies primarily on additions and multiplications—operations inherently supported by many homomorphic encryption schemes—it is mathematically feasible. Moreover, this approach is practically advantageous.

As genomic services transition to cloud-based platforms, ensuring data security is crucial. The privateHLA framework safeguards sensitive genetic information while maintaining imputation accuracy comparable to plaintext methods. It offers computational and memory efficiency suitable for practical applications. Recently, international efforts to aggregate limited HLA reference panels aim to improve imputation accuracy across diverse ancestries.^56^^,^^57^^,^^58^^,^^59^^,^^60^^,^^61^^,^^62^ In addition, some of these services are also available via web-based platforms, facilitating more convenient access for researchers. By integrating homomorphic encryption with these initiatives, secure data sharing and pooling can be achieved. This contributes to comprehensive international HLA imputation panels and advances global immune-related genomic research.

### Limitations of the study

Despite the advantages of privateHLA, several limitations remain. First, the use of homomorphic encryption inherently results in increased computational time, especially during operations such as the TOP2 comparison function. Second, since privateHLA returns only the indices of imputed alleles, it is challenging to provide statistical measures such as *p*-values or confidence intervals, which are commonly used in downstream analyses. Third, the use of homomorphic encryption restricts the model choices to relatively simple algorithms (such as logistic regression in privateHLA), preventing the application of more sophisticated or complex models that might improve imputation accuracy. Finally, introducing new SNPs requires the model to be retrained from scratch.

## Resource availability

### Lead contact

For additional information, resources, and reagents, please contact the lead researcher, Buhm Han (buhm.han@snu.ac.kr), who will address all inquiries.

### Materials availability

This study did not generate new materials.

### Data and code availability

• This study utilizes existing public datasets, including the T1DGC panel, HAN Chinese panel, HapMap European panel, and Korean panel. Access to the T1DGC dataset requires a formal request through the designated DOI link (T1DGC Data: https://doi.org/10.58020/qrdt-eh40). The other datasets are freely available to the public via the links provided in the key resources table.
• All original code has been deposited at GitHub (https://github.com/SNUCP/privateHLA) and is publicly available as of the date of publication. Additional software used in this study, including Python, R, and other analysis tools, are listed in the key resources table with their respective sources.
• Any additional information required to reanalyze the data reported in this paper is available from the lead contact upon request.

## Acknowledgments

This work was supported by the 10.13039/501100003725National Research Foundation of Korea (NRF) (Grant numbers RS-2025-00553579 and RS-2023-00211649) funded by the Korean government, 10.13039/501100014188Ministry of Science, and ICT. This work was also supported by the Creative-Pioneering Researchers Program funded by 10.13039/501100002551Seoul National University and by the AI-Bio Research Grant through 10.13039/501100002551Seoul National University.

## Author contributions

H.K., I.H., Y.S. and B.H. conceptualized and designed the study. H.K. and I.H. conducted the investigation. H.K. performed formal analysis of the data in an unencrypted state. I.H. and Y.S. designed the algorithms for encrypted computation. I.H. developed the software, implementing homomorphic encryption-based code. H.K., I.H. and B.H. wrote the original draft of the manuscript. B.H. reviewed and edited the manuscript. All authors approved the final version of the manuscript.

## Declaration of interests

Buhm Han is the CEO of SpintoAI Inc.

## Declaration of generative AI and AI-assisted technologies in the writing process

During the preparation of this work the authors used Paiper-AI in order to refine the wording. After using this tool, the authors reviewed and edited the content as needed and take full responsibility for the content of the publication.

## STAR★Methods

### Key resources table

**Table:** 

REAGENT or RESOURCE	SOURCE	IDENTIFIER
**Deposited data**		

T1DGC panel	Mychaleckyj et al.^25^	https://doi.org/10.58020/qrdt-eh40
HAN Chinese panel	Zhou et al.^26^	http://gigadb.org/dataset/100156
HapMap European panel	de Bakker et al.^23^	https://software.broadinstitute.org/mpg/snp2hla/
Korean panel	Kim et al.^24^	https://journals.plos.org/plosone/article?id=10.1371/journal.pone.0112546

**Software and algorithms**		

privateHLA software	This paper	https://github.com/SNUCP/privateHLA
Python	Oliphant^63^	https://www.python.org
R	Ihaka and Gentleman^64^	https://www.r-project.org
tfhe-go	Hwang^46^	https://github.com/sp301415/tfhe-go
Go	The GO Authors	https://go.dev
Beagle	Browning and Browning^65^	https://faculty.washington.edu/browning/beagle/b3.html

### Experimental model and study participant details

This study is a computational analysis based on existing, publicly available datasets and does not involve the use of experimental models typical in the life sciences.

### Method details

#### TFHE scheme

We provide a brief overview of TFHE (Torus Fully Homomorphic Encryption), the main encryption scheme used in privateHLA. TFHE is a type of encryption that allows computations to be performed directly on encrypted data, without the need to first decrypt it. This enables sensitive data to remain secure and private throughout the entire computation process. The main components of TFHE are as follows.

##### TFHE.KeyGen (Key Generation)

This procedure generates two keys: a secret key (sk), and a bootstrapping key (bk). The secret key is kept private and is used for encryption and decryption. The bootstrapping key, which is the public key in Figure 1, allows encrypted data to be processed repeatedly. It helps remove the extra noise that builds up each time a computation is done on encrypted data, which keeps the results accurate and usable.

##### TFHE.Enc (Encryption)

This process takes a plaintext and uses the secret key to convert it into an encrypted form called a ciphertext.

##### TFHE.Dec (Decryption)

After computations are performed on encrypted data, the ciphertext can be decrypted using the secret key.

##### TFHE.Bootstrap (Bootstrapping)

Every time a computation is done on encrypted data, a little bit of noise is added. If too much noise accumulates, the ciphertext becomes undecryptable. Bootstrapping is a special operation that cleans up this noise, making the encrypted data usable again. In TFHE, bootstrapping can also perform arbitrary function evaluation, while refreshing the data. This powerful feature, called programmable bootstrapping, makes it possible to carry out complex computations entirely on encrypted data.

However, the details of bootstrapping are not the primary focus or challenge addressed by privateHLA, so it is excluded from further detailed discussion in this work.

#### Functions of privateHLA

We assume the outsourced server has trained a logistic regression model for each HLA allele using plaintext training data, prior to processing the uploaded encrypted data. For each HLA allele, the regression model uses SNP genotypes (0, 1, and 2) as independent variables and a binary indicator of allele presence (absent and present) as a dependent variable. After the user uploads encrypted SNP data, the server calculates the encrypted probability of each HLA allele while preserving encryption, using trained weights. Then, the server returns only the most probable allele pairs for each gene in encrypted form, instead of sending all allele probabilities. Providing the probabilities of all alleles could lead to reconstruction attack or membership inference attack. Instead of revealing the probabilities of all possible allele pairs, privateHLA sequentially performs four main functions within the homomorphic encryption framework to return only the two most probable encrypted allele indices for each gene, thereby preserving privacy.

privateHLA consists of four functions: the Linear Function (*y =* $\hat{W}$ *x*), Top2 Function, Sigmoid Function, and the Thresholding Function (Figure S2). In the Linear Function, the server linearly combines encrypted SNP genotypes with the trained weights for each HLA allele. In the Top2 Function, the server identifies the two largest combined values as candidates for an allele pair for an HLA gene. The two largest values are then processed by the Sigmoid Function. The Sigmoid Function is applied to normalize the outputs of the Linear Function onto a consistent probability scale, ensuring stability for the Thresholding Function. Without it, the raw inner product values range from negative to positive, making it difficult to apply the Thresholding Function effectively. The Thresholding Function determines the genotype, assessing whether the HLA alleles are heterozygous or homozygous, using a threshold parameter from the validation dataset. Throughout these functions, all variables, except for the weights of the Linear Function, remain encrypted within homomorphic encryption, ensuring that the outsourced server cannot access the user’s information.

#### Threshold parameter for determining heterozygous or homozygous alleles

Before applying the four functions within homomorphic encryption, a threshold parameter (t) in the Thresholding Function is set using the validation set in plaintext. After computing probabilities of all alleles for a sample, the two highest probabilities, $p_{f}$ and $p_{s}$, are identified. Then, the genotype is determined as:

$$\left(a_{1},a_{2}\right)=\left\{ \begin{array}{l} \left(a_{f},a_{f}\right)\;for\;p_{f}\geq p_{s}\times t\;\\ \left(a_{f},a_{s}\right)\;for\;p_{f}<p_{s}\times t. \end{array}\right.$$

Here, $\left(a_{1},a_{2}\right)$ represent the imputed allele pair. The Thresholding Function plays a crucial role by returning the indices of the selected allele pair based on the optimal t, ensuring the highest accuracy. The optimal t is chosen from the range t∈{2,3,...,20}, with 15 selected for the Korean panel and 17 for the HapMap European panel. These selected values, determined in plaintext, are then applied to classify the target panel’s alleles within homomorphic encryption.

#### Linear function in homomorphic encryption

During encryption, we pack multiple SNPs into a GLWE ciphertext, similar to Chimera.^11^ In our parameters, we can pack 2048 SNPs into a single ciphertext. If the number of SNPs exceeds this limit, we pack them into multiple ciphertexts. Similarly, we also pack regression coefficients into GLWE plaintexts. Then, by utilizing plaintext-ciphertext multiplication, we can compute the linear combination between weights and SNPs.

#### Top2 function in homomorphic encryption

TFHE scheme originally supports only integer operations, whereas SNPs and weights are real numbers. To solve this problem, we use a CKKS-style encoding, where the input values are multiplied with some large number, called scale, then rounded to nearest integer. We choose the scale appropriately so that the values don’t overflow. After computing the Linear Function, we normalize the values using the function defined as follows.

$$normalize\left(x\right)=\left\{ \begin{array}{c} \frac{Q}{4}\cdot \frac{x-T}{B-T}\;if\;x\geq T\\ 0\;if\;x\leq T \end{array}\right.$$

Here, B is the expected bound for x, and T is some parameterized threshold value for normalization. As a result, the output values are normalized to $\left(0,\frac{Q}{4}\right)$ where Q is the ciphertext modulus, which is crucial for later steps.

To implement Top2 Function over fully homomorphic encryption (FHE), we compute the max function homomorphically. Given two ciphertexts Enc(a) and Enc(b), we first compute the following cmpbit function over Enc(a)−Enc(b).

$$cmpbit\left(x\right)=\left\{ \begin{array}{l} \frac{Q}{2}\;if\;x\geq 0\\ 0\;if\;x<0 \end{array}\right.$$

In other words, ${ct}_{c}=cmpbit\left(Enc\left(a\right)-Enc\left(b\right)\right)$ encrypts Q/2 if a≥b, and 0 otherwise. Using this ciphertext, we can compute $\max\left(Enc\left(a\right),Enc\left(b\right)\right)=mux\left(ct_{c}+Enc\left(a\right)\right)+mux\left(\frac{Q}{2}-ct_{c}+Enc\left(b\right)\right)$, where mux is defined as follows.

$$mux\left(x\right)=\left\{ \begin{array}{c} x-\frac{Q}{2}\;if\;x\geq \frac{Q}{2}\\ 0\;if\;x<\frac{Q}{2} \end{array}\right.$$

The correctness can be shown as follows. Suppose a≥b. Then, $ct_{c}$ encrypts $\frac{Q}{2}$, and $ct_{c}^{\prime }=\frac{Q}{2}-ct_{c}$ encrypts 0. Since we normalized a,b to be smaller than $\frac{Q}{4}$, $mux\left(ct_{c}+Enc\left(a\right)\right)=mux\left(Enc\left(\frac{Q}{2}+a\right)\right)=Enc\left(a\right)$, and $mux\left(ct_{c}^{\prime }+Enc\left(b\right)\right)=mux\left(Enc\left(b\right)\right)=Enc\left(0\right)$ we have max(E(a),E(b))=E(a) as desired. The case where a<b can be shown similarly. Note that we can also compute min(Enc(a),Enc(b))=Enc(a)+Enc(b)−max⁡(Enc(a),Enc(b)).

#### Sigmoid Function in homomorphic encryption

After selecting the top 2 ciphertext, we compute the Sigmoid Function using programmable bootstrapping in TFHE. Note that we need to reverse the normalization function in the Linear Function step. As a result, we compute the following function homomorphically.

$$sigmoid^{\prime }\left(x\right)=sigmoid\circ {normalize}^{-1}\left(x\right)$$

#### Thresholding Function in homomorphic encryption

In the Thresholding Function step, we take a similar approach as in the Top2 Function. First, to prevent overflowing, instead of comparing $p_{f}$ and $p_{s}\times t$, we compare $\frac{p_{f}}{t}$ and $p_{s}$ by homomorphically dividing the encryption of $p_{f}$ by t. Then, we compute $ct_{c}=cmpbit\left(Enc\left(\frac{p_{f}}{t}\right),Enc\left(p_{s}\right)\right)$. Finally, we can compute $Enc\left(a_{2}\right)=mux\left(ct_{c}+Enc\left(p_{f}\right)\right)+mux\left(\frac{Q}{2}-ct_{c}+Enc\left(p_{s}\right)\right)$, similar to the Top2 Function step. Note that $a_{1}$ always equals $p_{f}$, so no further operation is needed for it.

#### Data preparation

We utilized the Han Chinese^26^ and T1DGC^25^ reference panels to impute the Korean^24^ and HapMap European^23^ target panels respectively. The Han Chinese panel contained no missing values, whereas the other panels did. We first imputed the missing SNPs in the reference panel (T1DGC) using Beagle.^65^^,^^66^ Next, we performed quality control by extracting SNPs against the HRC, 1000 Genomes, and CAAPA SNP lists^67^^,^^68^^,^^69^ on the reference panels (Han Chinese and T1DGC). Finally, we imputed the target panels using Beagle again, employing their corresponding reference panels. As a result, we obtained 5,754 SNPs common to both the T1DGC and the HapMap European panels, and 21,820 SNPs common to both the Han Chinese and Korean panels. Because we need a validation dataset to determine threshold parameters, we split the reference panels into training and validation datasets. Specifically, the T1DGC panel was divided into 1,000 validation samples and 4,225 training samples, while the Han Chinese panel was divided into 1,000 validation samples and 8,773 training samples. The details are as follows.

##### Korean panel

The Korean panel^24^ includes *HLA-A*, *-B*, *-C*, *-DPB1*, *-DQB1* and *-DRB1*. HLA was genotyped using Roche’s GS 454 sequencing at the Institute for Immunology and Infectious Diseases, and called with algorithms certified by the American Society for Histocompatibility and Immunogenetics. The Korean panel consists of 5,619 SNPs and 413 individuals.

##### HapMap European panel

The HapMap European panel^23^ includes *HLA-A*, *-B*, *-C*, *-DQA1*, *-DQB1* and *-DRB1*. HLA was genotyped by PCR-SSOP-based protocols. We downloaded the data for 124 individuals from the SNP2HLA website,^4^ retaining 88 unrelated individuals. The final HapMap European panel consists of 4,290 SNPs and 88 individuals.

##### HAN Chinese panel

The HAN Chinese panel^26^ includes *HLA-A*, *-B*, *-C*, *-DPA1*, *-DPB1*, *-DQA1*, *-DQB1*, *-DRB1*, *-DRB3*, *-DRB4* and *-DRB5*. However, we didn’t use *HLA-DRB3*, *-DRB4* and *-DRB5* in this study. HLA was genotyped by the targeted NGS sequencing and in-silico typing software.^70^ We downloaded the data from the website described in Zhou et al.^26^ We removed individuals lacking exactly two appearances of the “presence” in the binary markers. The final HAN Chinese panel used in this study consists of 27,780 SNPs and 9,773 individuals.

##### T1DGC panel

The T1DGC panel^25^ includes *HLA-A*, *-B*, *-C*, *-DPA1*, *-DPB1*, *-DQA1*, *-DQB1* and *-DRB1*. HLA was genotyped by SSOP technology, involving PCR amplification of exon 2 and 3 in Class I and exon 2 in Class II. We downloaded the data from the SNP2HLA website.^4^ The T1DGC panel used in this study consists of 8,961 SNPs and 5,225 individuals.

#### Non-secure methods

##### SNP2HLA

SNP2HLA^4^ is a method for HLA genotype imputation, utilizing Beagle v3^66^ software which is a hidden Markov model. SNP2HLA treats the multi-alleles of HLA genes as individual binary alleles for imputation. SNP2HLA is capable of imputing genotypes for class I and II HLA loci, along with amino acid polymorphisms. A notable advantage of SNP2HLA is its ability to simultaneously impute HLA genotypes and amino acid allele genotypes.

##### CookHLA

CookHLA^5^ is an expansion of SNP2HLA. CookHLA uses more recent versions of the Beagle software, specifically v4 and v5,^65^^,^^71^ unlike SNP2HLA which relies on Beagle v3. Another improvement is CookHLA's ability to capture information across each exon, providing a broader local context for more precise imputation. This contrasts with SNP2HLA's approach, which positions markers centrally within the HLA gene.

### Quantification and statistical analysis

All performance evaluations presented in this paper were conducted using Python and R. We measured the imputation accuracy of our proposed privateHLA method and compared it against established methods (SNP2HLA and CookHLA) to validate its performance.

We measured the imputation accuracy defined in CookHLA.^6^ In the test dataset, we define $\left(A_{1},{\;A}_{2}\right)$ as true pair and ($B_{1},{\;B}_{2})$ as predicted pair of alleles for an HLA gene. Then, we calculated the accuracy as the average value of the following.

$$\frac{\max\left(I\left(A_{1}=B_{1}\right)+I\left(A_{2}=B_{2}\right),I\left(A_{1}=B_{2}\right)+I\left(A_{2}=B_{1}\right)\right)}{2}$$

I is an indicator function that returns 1 if the alleles match and 0 otherwise. We calculated accuracy measurements using two-field HLA alleles, grouping them into functionally indistinguishable protein sequences at the P-group level for evaluation, and excluded alleles whose names have been deprecated in the recent version of the IPD-IMGT/HLA database.^72^
