# Supplementary material for: A privacy-preserving HLA imputation method with homomorphic encryption
Source: iScience. 2025 Aug 26;28(9):113442. doi: 10.1016/j.isci.2025.113442 (PMC12496177; doi:10.1016/j.isci.2025.113442)
Supplement: Document S1. Figures S1 and S2 [file mmc1.pdf]

iScience, Volume *28*

Supplemental information

A privacy-preserving HLA imputation method with homomorphic encryption

Hakin Kim, Intak Hwang, Yongsoo Song, and Buhm Han

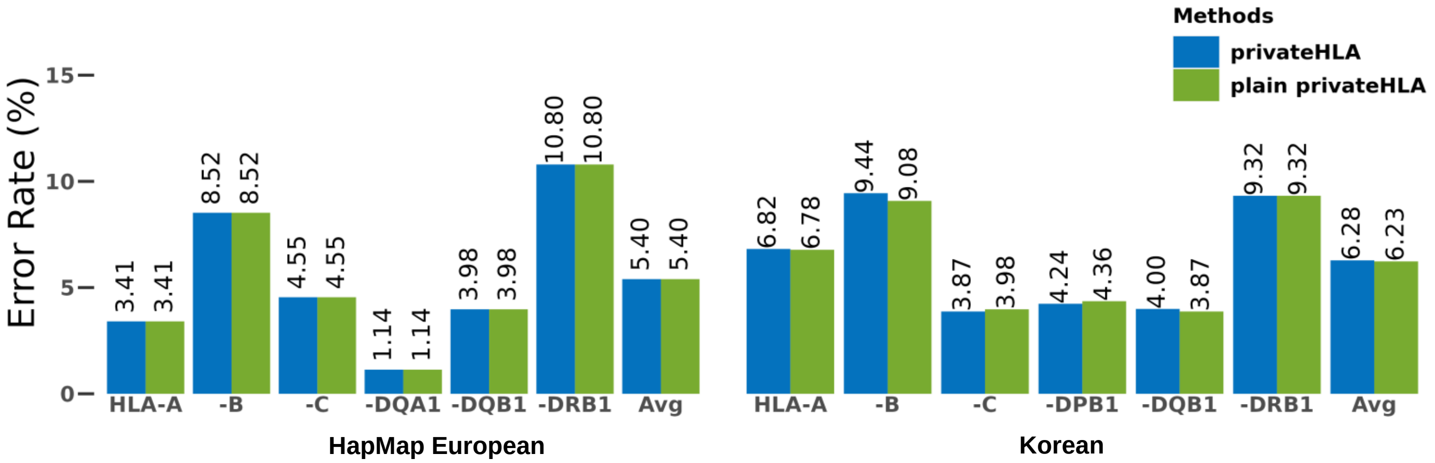

Figure S1. Error rate comparison between privateHLA (encrypted) and plain privateHLA (non-encrypted)
The results show negligible accuracy loss due to encryption in both HapMap European and Korean panels.

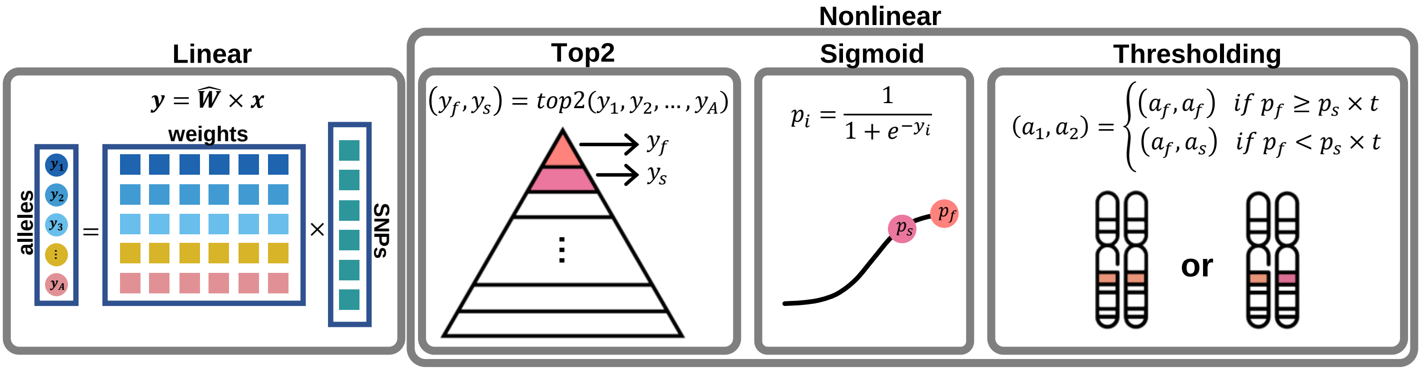

Figure S2. Functions in privateHLA

privateHLA consists of four main functions: one linear function and three nonlinear functions. The linear function calculates the weighted summation of SNP genotypes and their corresponding weights for each allele. The Top2 function compares the outputs of the linear function to identify the two highest values. The sigmoid function then maps these two values onto a probability scale between 0 and 1. Finally, the thresholding function determines whether the result is heterozygous or homozygous given a threshold parameter.
